# Classification of Twitter Vaping Discourse Using BERTweet: Comparative Deep Learning Study

**DOI:** 10.2196/33678

**Published:** 2022-07-21

**Authors:** William Baker, Jason B Colditz, Page D Dobbs, Huy Mai, Shyam Visweswaran, Justin Zhan, Brian A Primack

**Affiliations:** 1 Department of Computer Science and Computer Engineering University of Arkansas Fayetteville, AR United States; 2 Division of General Internal Medicine University of Pittsburgh School of Medicine Pittsburgh, PA United States; 3 Health, Human Performance and Recreation Department University of Arkansas Fayetteville, AR United States; 4 Department of Biomedical Informatics University of Pittsburgh Pittsburgh, PA United States; 5 College of Public Health and Human Science Oregon State University Corvallis, OR United States

**Keywords:** vaping, social media, deep learning, transformer models, infoveillance

## Abstract

**Background:**

Twitter provides a valuable platform for the surveillance and monitoring of public health topics; however, manually categorizing large quantities of Twitter data is labor intensive and presents barriers to identify major trends and sentiments. Additionally, while machine and deep learning approaches have been proposed with high accuracy, they require large, annotated data sets. Public pretrained deep learning classification models, such as BERTweet, produce higher-quality models while using smaller annotated training sets.

**Objective:**

This study aims to derive and evaluate a pretrained deep learning model based on BERTweet that can identify tweets relevant to vaping, tweets (related to vaping) of commercial nature, and tweets with provape sentiment. Additionally, the performance of the BERTweet classifier will be compared against a long short-term memory (LSTM) model to show the improvements a pretrained model has over traditional deep learning approaches.

**Methods:**

Twitter data were collected from August to October 2019 using vaping-related search terms. From this set, a random subsample of 2401 English tweets was manually annotated for relevance (vaping related or not), commercial nature (commercial or not), and sentiment (positive, negative, or neutral). Using the annotated data, 3 separate classifiers were built using BERTweet with the default parameters defined by the Simple Transformer application programming interface (API). Each model was trained for 20 iterations and evaluated with a random split of the annotated tweets, reserving 10% (n=165) of tweets for evaluations.

**Results:**

The relevance, commercial, and sentiment classifiers achieved an area under the receiver operating characteristic curve (AUROC) of 94.5%, 99.3%, and 81.7%, respectively. Additionally, the weighted F1 scores of each were 97.6%, 99.0%, and 86.1%, respectively. We found that BERTweet outperformed the LSTM model in the classification of all categories.

**Conclusions:**

Large, open-source deep learning classifiers, such as BERTweet, can provide researchers the ability to reliably determine if tweets are relevant to vaping; include commercial content; and include positive, negative, or neutral content about vaping with a higher accuracy than traditional natural language processing deep learning models. Such enhancement to the utilization of Twitter data can allow for faster exploration and dissemination of time-sensitive data than traditional methodologies (eg, surveys, polling research).

## Introduction

### Background

Since its launch in 2006, Twitter has exploded in popularity to become one of the top social media platforms. As of 2021, the site hosts 192 million daily active users worldwide [1]. The 280-character constraint on a Twitter text post, called a tweet, lends itself well to spontaneous and organic interactions. The candid nature of the tweets provides invaluable data for the public health realm. Patients spend relatively little time with health care professionals, with some only seeing their primary care physician once every other year, and therefore it can be difficult for health care workers to accurately address needs or feelings that patients often find uncomfortable disclosing to others [2].

While Twitter provides a valuable platform for the surveillance and monitoring of public health topics, manually categorizing large quantities of Twitter data by hand presents challenges to identify major trends and sentiments in a timely manner. Machine and deep learning methods have previously been proposed to provide a framework for systematic and automated processing and analysis of Twitter data to develop surveillance systems with applications to public health [3]. While these models achieve high accuracy, they require large sets of annotated data to be trained. By contrast, public pretrained deep learning classification models, such as BERTweet, produce higher-quality models while using smaller annotated training sets [4]. In this study, we derive and evaluate a pretrained deep learning model based on BERTweet that can identify tweets relevant to vaping, tweets of commercial nature, and tweets with provape sentiment. We compare the results of the BERTweet-based classifier with a long short-term memory model (LSTM) to show the improvements a pretrained model has over traditional deep learning approaches.

### Traditional Deep Learning

Deep learning is a class of machine learning algorithms that uses multiple layers to progressively extract higher-level features from raw input [4]. Several types of deep learning architectures exist, such as deep neural networks, recurrent neural networks (RNNs), and convolutional neural networks (CNNs). Applications of deep learning include computer vision, speech recognition, natural language processing, and drug design.

In their work, Visweswaran et al [3] found that LSTM models performed particularly well on tweet classification for relevance, sentiment, and commercial nature [3]. An LSTM network is a special kind of RNN capable of learning long-term dependencies [5]. Unlike standard feedforward networks, such as CNNs, LSTMs have a feedback connection. This feedback connection allows the network to not only process a single data point (ie, a word), but also entire sequences of data (ie, sentence or phrase), which make them extremely powerful in classifying sentiment of a message.

### Pretrained Transformer Models

Over the last few years, transformer models have been very effective for a large variety of natural language processing tasks. First proposed by Colditz et al [6], transformers use a self-attention mechanism to capture what aspects of a sequence are important in a series of tokens. In simple terms, self-attention mechanisms aim to create real natural language understanding in machines.

In 2018, Google AI Language released the Bidirectional Encoder Representations from Transformers (BERT) model, which improves upon the original transformer model by learning token representations in both directions [7]. In normal transformers, a sequence is analyzed either left to right or right to left, but not in both directions. To achieve this, BERT uses a revamped pretraining procedure that includes masked language model and next sentence prediction objectives [2]. Several BERT models pretrained on a variety of texts, languages, and topics are available freely to the public. This creates a ready-made approach for researchers trying to create models for a number of language tasks, including text classification. Researchers can use BERT in its default settings, or they can apply fine-tuning on a data set closely applicable to the task at hand. For instance, in this study, the created model is fine-tuned on a set of hand-annotated tweets before testing the classification accuracy of the system.

After BERT was introduced, the “Robustly optimized BERT pre-training approach” (RoBERTa) was published [8]. RoBERTa was created out of the authors’ experimentation with the default hyperparameters of BERT. They found that BERT was significantly undertrained, and that with some minor changes, the modified BERT model was able to outperform newer and even larger transformer models. Pretraining optimizations in RoBERTa include dynamic masking, large mini-batches, larger byte-pair encodings, and using full sentences across documents. We refer to Liu et al [8] for a more detailed discussion of the optimizations performed in RoBERTa. Like BERT, many pretrained variations of RoBERTa are available online.

BERTweet is a public BERT-based model trained using the RoBERTa pretraining procedure [9]. Released in 2020, it was the first large-scale pretrained language model for English tweets to be released to other researchers for further improvements and novel applications. BERTweet was trained on 850 million English tweets collected from 2012 to 2019, which prepares it well for novel downstream classification tasks on a set of tweets. This pipeline of pretraining on a large text corpus and then fine-tuning the model for classification tasks is called transfer learning [2]. It has been shown that pretraining is integral to model performance on downstream tasks, and it follows that pretraining a model on material that is similar to the texts in the downstream task will yield improved performance. Therefore, having access to a model trained on a large corpus of tweets is invaluable for the creation of a Twitter-based public health surveillance system. We refer to Nguyen et al [9] for a more detailed explanation of how the BERTweet model functions.

### Objective

It is our goal to produce an accurate BERTweet-based deep learning classifier that can improve upon existing Twitter surveillance systems that are focused on vaping-related tweets. Additionally, we aim to produce a classifier that is reliable and accurate in assessing a tweet for relevance (relevant or not), sentiment (positive, negative, or neutral), and commercial nature (commercial or not). Leveraging Twitter as a complement to traditional surveillance will allow for real-time identification of changes that can be used by public health practitioners. For example, when positive sentiment toward vaping rises, practitioners may be able to determine the exact reasons why and respond accordingly. Similarly, when there is a notable spike in misinformation about vaping and its effects on health, health experts will be able to act immediately to correct this information [3].

### Related Work

Several works have proposed classifiers to classify Twitter data in terms of sentiment. Further, the last few years have seen a surge in publications on creating classifiers to analyze public health trends as depicted on Twitter. Gohil et al [10] performed a review of current sentiment analysis tools available for researchers. They found that while multiple methods existed for analyzing the sentiment of tweets in the health care setting, there is still the need for an accurate and verified tool for sentiment analysis of tweets trained using a health care setting–specific tweet. Edara et al [11] developed an LSTM to classify cancer-related tweets based on the tone of the tweet and compared the results against several traditional machine learning approaches. They found that the LSTM model outperformed all of the other approaches. Ji et al [12] utilized the Twitter platform to monitor the spread of public concern about epidemics by separating personal tweets from new tweets and then further categorizing the personal tweets into those that are negative and nonnegative using a naïve Bayes classifier.

For a general approach to performing a sentiment analysis on Twitter data, Agarwal et al [13] introduced unigram, feature-based, and tree-based models to classify tweets as either a binary task (positive or negative) or a 3-way task (positive, negative, and neutral). Harjule et al [14] proposed another general approach to classifying the sentiment of tweets. The authors analyzed several lexicon and machine learning–based tweet sentiment classifiers on a large group of data sets and found that the machine learning models were more accurate at classifying sentiment. Kharde and Sonawane [15] performed a similar comparative analysis and verified the claim from Harjule et al [14] that machine learning classifiers yield higher accuracy, with the caveat that lexicon-based methods can be more affective in some cases.

Beyond general sentiment and public health monitoring, several studies have looked at using Twitter to monitor trends toward vaping and e-cigarettes [16,17]. Han and Kavuluru [18] implemented several machine learning models, such as support vector machines, logistic regression, and CNNs, to identify marketing and nonmarketing e-cigarette tweets. Further, Myslín et al [19] and Cole-Lewis et al [20] annotated tobacco-related tweets and derived several machine learning classifiers to predict sentiment. Huang et al [21] analyzed tweets using machine learning classifiers to find trend in the commercial nature of tweets relating to vaping. They found that tweets related to e-cigarettes were about 90% commercial and about 10% mentioned smoking cessation. Resende and Culotta [22] derived a sentiment classifier for e-cigarette–related tweets that identified positive and negative tweets with 96% and 70% precision, respectively. Visweswaran et al [3] performed an in-depth comparison of traditional machine learning classifiers (regression, random forest, linear support vector machine, and multinomial naïve Bayes) with deep learning classifiers (CNN, LSTM, LSTM-CNN, and bidirectional LSTM), and found that among all the tested networks, LSTM achieved the highest classification accuracy.

## Methods

### Data Collection

Tweets were collected continuously from August to October 2019 using the Real-Time Infoveillance of Twitter Health Messages (RITHM) framework [6]. The RITHM framework is an open-source software for collecting and formatting Twitter data. It additionally provides procedures for maximizing the efficiency and effectiveness of subsequent human data coding. The keywords that we used for data collection include *vape, vapes, vaper, vapers, vaping, juul, juuls,* and *juuling*. The vaping-related keywords are based on previous Twitter research [6,10] and, in particular, we included keywords to identify the highly popular e-cigarette brand, JUUL, which had the highest market share at the time from which data were collected [23]. We identified and collected all tweets that matched 1 or more keywords from the list above.

### Annotation

After data collection, a random subsample of 2401 English tweets was annotated for relevance (vaping related or not), commercial nature (commercial or not), and sentiment (positive, negative, or neutral). This annotation was done in accordance with the 3-level hierarchical annotation schema, as depicted in Table 1. A tweet was first annotated for relevance. Then, only if the tweet was relevant, was it annotated for commercial nature and sentiment.

A team of 2 trained annotators independently annotated batches of 400 tweets at a time. Adjudicated annotation disagreements were carried out under the presence of the supervising investigator. All annotates codes have a Cohen κ value over 0.70, indicating strong internal agreement among annotators. The full set of 2401 adjudicated annotations and tweet content were used in the training of the classifier models. A detailed description of the annotations can be found in Table 2.

**Table 1:** Descriptions of labels used for annotating vaping-related tweets.

Labels	Descriptions	Example quotes
Relevant	Is the tweet in English and related to the vaping topic at hand (eg, vape use or users, vaping devices, or products)?	
Not relevant	Typically, non-English tweets or tweets that referenced vaping cannabis products specifically.	
Commercial	Is the tweet an advertisement/marketing for vaping products?	*Today only! Buy one JUUL get the second half price with our online coupon code #JUUL4LIFE*
Noncommercial	Includes tweets that demonstrate favorability toward a product but do not directly advocate for purchasing it.	
Positive	The tweet is associated with positive emotions or contexts regarding vaping.	The tweeter is currently, or has recently used, or is going to vape: *Currently juuling in the bathroom at school!* The tweeter shows positivity or neutral acceptance from others’ usage or others’ positive comments about vaping: *Just got Hannah to try vaping for the first time! She loved it.* The tweeter mentions a vape pen in association with other positive aspects of society or popular culture. *We need a Disney princess that rips her JUUL in the middle of a serious conversation.* The tweeter asks a question using first-person pronouns: *Where can I buy a JUUL?*
Negative	The tweet is associated with negative emotions or contexts regarding vaping.	The tweeter believes smoking a vape is disgusting, uncool, or unattractive: *Cannot believe everyone is smoking JUULs these days. I think it’s disgusting.* The tweeter criticizes/ridicules others for using a vape: *ur mcm says ‘cigarettes are gross’ yet is addicted to nicotine through cool cucumber flavored JUUL pods.* The tweeter prefers to use a different substance, such as cigarettes or marijuana: *Tried a JUUL today for the first time but I still prefer cigarettes over it.*
Neutral		The tweet is factual but not opinionated or is a question about unbiased facts/information about vaping: *They are selling JUUL pens at my local tobacco shop for anyone interested.* *What is a JUUL?* *Is a JUUL better than tobacco?*

**Table 2:** Description of annotated training and test data sets (N=2401).^a^

Targets	Number of tweets with a positive target, n (%)	Number of tweets with a negative target, n (%)	Number of tweets with a neutral target, n (%)
Relevance	Relevant: Total: 1802 (75.05) Training: 1637 (90.84) Test: 165 (9.16)	Nonrelevant: Total: 599 (24.95) Training: 524 (87.48) Test: 75 (12.52)	N/A^b^
Commercial	Commercial: Total: 117 (4.87) Training: 106 (90.60) Test: 11(9.40)	Noncommercial: Total: 1685 (70.18) Training: 1516 (89.97) Test: 169 (10.03)	N/A
Sentiment	Positive: Total: 172 (7.16) Training: 158 (91.86) Test: 14 (8.14)	Negative: Total: 130 (5.41) Training: 119 (91.54) Test: 11 (8.46)	Neutral: Total: 1372 (57.14) Training: 1229 (89.58) Test: 143 (10.42)

^a^Percentages may not add up to 100% as classification was made for sentiment only if the tweet was relevant.

^b^Sentiment-only code with neutral target.

### LSTM Model

We will briefly recount the process explained in Visweswaran et al [3] to train and evaluate an LSTM model to classify a tweet related to vaping as relevant; commercial; and if it was positive, negative, or neutral in sentiment. Our LSTM model was developed using the built-in functionality of the TensorFlow machine learning library. We utilized rectified linear unit (ReLU) as the activation function of the hidden layers and the sigmoid activation function for the output layer. Additionally, we utilized binary cross entropy as the loss function with Adam as the optimizer. In accordance with Visweswaran et al’s study [3], we used nondomain-specific GloVe word vectors.

After first testing a 70/30 split to create the relevance classifier and testing random splits to prevent over fitting, we found optimal results with a 90/10 split of the entire annotated data set, as all tweets were coded as either relevant or nonrelevant. We used the 90% split (n=1637) to train the LSTM relevance classifier, and then tested on the remaining 10% (n=165). We trained the model for 5 epochs using a batch size of 64. Both the commercial and sentiment classifiers followed the same training and testing procedures as the relevance classifier. The one difference being that only tweets labeled as relevant were used in the commercial and sentiment data sets. All nonrelevant tweets were filtered out and discarded.

### BERTweet

To create a classifier for relevance, 90% of the tweets labeled as either relevant (n=1637) or nonrelevant (n=524) were used to fine-tune the BERTweet model, and the remaining 10% were used to test the final model (relevant n=165; nonrelevant n=75). This splitting, training, and testing process was repeated multiple times with random splits, and the accuracy results are the averages of each individual run. BERTweet was trained for 20 epochs with a batch size of 32 and a learning rate of 5 × 10^–5^. All other hyperparameters were left to the default values according to Simple Transformers API, which was used to accelerate the fine-tuning process for BERTweet and decrease the amount of proprietary code needed to be written. Tokenization of input tweet text was handled by Simple Transformers API, which automatically uses the BERTweet tokenizer defined by the creators of the model.

To create the commercial and sentiment classifiers, annotated tweets were first filtered by relevance; nonrelevant tweets were discarded for these classifiers, and tweets marked relevant were then split into training and testing sets, and models were fine-tuned using the same process as the relevance classifier.

## Results

### Overview

We compared the performance of the LSTM and BERTweet classifiers in terms of F1 and AUROC scores. Additionally, each score is the average of 3 different testing iterations of the respective models. F1 is a function of precision and recall:

F1 = 2×(Precision × Recall)/(Precision + Recall) (1)

Precision = True positive/(True positive + False positive) (2)

Recall = True positive/(True positive + False negative) (3)

For F1, values closer to 1 on a scale of 0 to 1 indicate good balance between precision and recall.

AUROC is the measure of the discrimination of the models, that is, for example, how well a classifier differentiates between positive, negative, and neutral tweets. The larger the AUROC score is, the better the model performs.

### Relevance

With regard to classifying a tweet as relevant or nonrelevant, the BERTweet classifier obtained an F1 score of 0.976 and an AUROC score of 0.945. The LSTM classifier achieved an F1 score of 0.924 and an AUROC score of 0.924. All runs of the BERTweet classifier achieved higher F1 and AUROC scores than the LSTM model.

### Commercial

In classifying commercial tweets (commercial or noncommercial) the BERTweet classifier performed well with an F1 score of 0.990 and an AUROC of 0.993. Of all classes, the BERTweet performed best in commercial classification. The LSTM model produced a lower F1 score of 0.727 and a lower AUROC score of 0.903 in comparison to the BERTweet model (Table 3).

**Table 3:** Comparison of BERTweet and LSTM^a^ F1 and AUROC^b^ scores.

Classifier/metric			Relevance		Commercial	Sentiment
**BERTweet**						
	F1	0.976		0.990		0.861
	AUROC	0.945		0.993		0.817
**LSTM**						
	F1	0.924		0.727		0.250
	AUROC	0.924		0.903		0.776

^a^LSTM: long short-term memory.

^b^AUROC: area under the receiver operating characteristic curve.

### Sentiment

Both the BERTweet and LSTM models performed the worst in the classification of sentiment (positive, negative, or neutral). BERTweet obtained an F1 of 0.861 with an AUROC of 0.817. The LSTM model had an F1 of 0.250 with an AUROC of 0.776.

## Discussion

### Principal Findings

This is the first study to use BERTweet to classify vaping-related tweets. Based on the analyses, we found that pretrained deep learning classifiers such as BERTweet perform exceptionally well at classifying a tweet as being relevant to vaping, being a commercial-natured tweet about vaping, as well as the sentiment of a tweet toward vaping. Compared with the LSTM classifier, the BERTweet classifier had AUROC values of 0.945, 0.993, and 0.817 for relevance, commercial nature, and sentiment, respectively. In general, these results show that pretrained classifiers can be utilized to monitor social medial platforms such as Twitter for public health trends. Such enhancement to the utilization of Twitter data can allow for faster exploration and dissemination of time-sensitive data than traditional methodologies such as surveys and polling research.

Practically, our work also serves to provide public health practitioners with vaping-related information on Twitter. For example, if there is an increase in positive sentiments of tweets, public health practitioners may find that a particular area is ready for policy change. Using the classification results, practitioners can also understand how many tweets are related to marketing of vaping and the relationship between sentiment of people and number of commercial tweets.

### Limitations

This study was performed with several limitations. First, a relatively small set of 2401 tweets was annotated by hand. Compared with another study [3], this was just over half the size of the data set they annotated. While the set was small, it was enough to produce accurate results when using BERTweet, which is another testament to the power that pretrained transformer models have. However, this limitation does make it difficult to compare results directly with Visweswaran et al [3]. Second, while we matched keywords with Visweswaran et al’s study [3], due to the evolving nature of language on Twitter, our collection methods could have overlooked new products or trends that have become prevalent on the Twitter platform. Third, we analyzed tweets that were written in English only. This limits the populations from which this classifier can accurately classify tweets. For instance, other countries may have different sentiments toward vaping that were not supported in this study. Finally, the date range of the tweets was limited to a 2-month time span, which limits the generalizability of the classifier over time, and therefore, more analysis would need to be performed to discover the longevity of the classifier.

### Future Research

Several different research endeavors relating to utilizing pretrained deep learning models to classifying tweets could be explored. First, we could expand from analyzing only English tweets to diversify this work for global regions and languages. Additionally, analysis on the number of annotated tweets needed to create an equivalent LSTM model could be performed to give substantial evidence that pretrained models provide evidence just beyond higher classification accuracy. Finally, the BERTweet model developed in this paper could be extended to create a real-time analysis platform for sentiment toward vaping to better inform public health officials, allowing them to understand the impacts of current and future policy interventions.

### Conclusion

In this study, we produced a deep learning classification model based on BERTweet that was able to classify a vaping-related tweet along several viewpoints such as relevance (relevant or not), commercial nature (commercial or not), and sentiment (positive, negative, or neutral). We then compared the classification performance of the BERTweet model with that of an LSTM model for the classification of 2401 hand-coded tweets. We found that in all classification cases BERTweet achieved higher levels of accuracy. The strong performance of BERTweet shows that it can increase the ability to accurately monitor social platforms such as Twitter with regard to public health trends such as vaping.

## Abbreviations

API: application programming interface
AUROC: area under the receiver operating characteristic curve
BERT: bidirectional encoder representations from transformer
CNN: convolutional neural network
LSTM: long short-term memory
ReLU: rectified linear unit
RITHM: Real-time Time Infoveillance of Twitter Health Messages
RNN: recurrent neural network
RoBERTa: robustly optimized BERT pre-training approach
